# Dosimetric Impact on the Flattening Filter and Addition of Gold Nanoparticles in Radiotherapy: A Monte Carlo Study on Depth Dose Using the 6 and 10 MV FFF Photon Beams

**DOI:** 10.3390/ma15207194

**Published:** 2022-10-15

**Authors:** Armando Spina, James C. L. Chow

**Affiliations:** 1 Department of Physics, Toronto Metropolitan University, Toronto, ON M5B 2K3, Canada; 2 Radiation Medicine Program, Princess Margaret Cancer Centre, University Health Network, Toronto, ON M5G 1X6, Canada; 3 Department of Radiation Oncology, University of Toronto, Toronto, ON M5T 1P5, Canada

**Keywords:** nanomaterials, gold nanoparticles, gold NP-enhanced radiotherapy, Monte Carlo simulation, flattening filter, flattening filter-free photon beam and dose enhancement

## Abstract

Purpose: This phantom study investigated through Monte Carlo simulation how the dose enhancement varied with depth, when gold nanoparticles (NPs) were added using the flattening filter-free (FFF) photon beams in gold NP-enhanced radiotherapy. Method: A phantom with materials varying from pure water to a mixture of water and gold NPs at different concentrations (3–40 mg/mL) were irradiated by the 6 and 10 MV flattening filter (FF) and FFF photon beams. Monte Carlo simulations were carried out to determine the depth doses along the central beam axis of the phantom up to a depth of 40 cm. The dose enhancement ratio (DER) and FFF enhancement ratio (FFFER) were calculated based on the Monte Carlo results. Results: The DER values were found decreased with an increase of depth and increase of NP concentration in the phantom. For the maximum NP concentration of 40 mg/mL, the DER values decreased 6.9, 12, 4.6 and 7.2% at a phantom depth from 2 to 40 cm, using the 6 MV FF, 6 MV FFF, 10 MV FF and 10 MV FFF photon beams, respectively. The maximum DER values for the 6 MV beams were 1.08 (FF) and 1.14 (FFF), while those for the 10 MV beams were 1.04 (FF) and 1.07 (FFF). When the FF was removed from the linear accelerator head, the FFFER showed a more significant increase of dose enhancement for the 6 MV beams (1.057) than the 10 MV (1.031). Conclusion: From the DER and FFFER values based on the Monte Carlo results, it is concluded that the dose enhancement with depth was dependent on the NP and beam variables, namely, NP concentration, presence of FF in the beam and beam energy. Dose enhancement was more significant when using the lower photon beam energy (i.e., 6 MV), FFF photon beam and higher NP concentration in the study.

## 1. Introduction

External beam radiotherapy refers to the application of ionizing radiation produced by a medical linear accelerator to kill cancer cells through (deoxyribonucleic acid) DNA damage [1,2]. Radiosensitizers are used to make tumour cells more sensitive to radiotherapy. This is advantageous for enhancing cell death in tumour while reducing the possible side effects on normal tissue [3]. This prompted the use of nanomaterials such as gold nanoparticles (NPs) in radiotherapy as plausible tools to enhance accuracy of localizing ionization, sparing healthy cells, while serving as a tool to enhance cancer cell kill [4]. In recent studies on Monte Carlo simulation, gold NPs have been proven to be an excellent radiosensitizer, to provide exceptional dose enhancement by dose enhancement ratio (DER) calculations for cancers [5,6,7].

For conventional dose delivery technique in radiotherapy such as 3D-conformal radiotherapy, a flattening filter (FF) made of aluminium, iron, copper and/or tungsten is used to generate a flattened photon beam to produce a homogeneous dose distribution at the tumour. However, modern radiotherapy has utilized more advanced delivery techniques such as intensity-modulated radiotherapy and volumetric modulated arc therapy [8,9], based on beam segments generated by a multi-leaf collimator to create more conformal dose distribution without using a FF. This photon beam is characterized as FF-free (FFF) beam. Since FF removes a large portion of low-energy photons from the beam source in the accelerator head, FFF photon beam contains more low-energy photons and has a softer energy spectrum in comparison to the FF photon beam [10]. The main advantage of using a FFF photon beam is the increased dose rate in comparison to the FF photon beam. The increase in dose rate shortens the treatment time and reduces the intrafraction organ motion of the patient during the treatment [11]. FFF photon beam also reduces the out-of-field dose and exposure to healthy tissues outside the treatment field [12]. However, FFF photon beam contributes to a lower depth dose, as these low-energy photons would deposit dose in the build-up region of the patient causing a higher and less preventative surface dose in comparison to the FF beam [13]. In contrast, head scatter and leakage are significantly reduced for the FFF photon beam, lowering the surface dose delivered to the patient [14].

When gold NPs are added to the patient in radiotherapy, dose enhancement occurs as the compositional atomic number of the tumour increases. This is because the photoelectric cross-section is proportional to *Z^n^*, where *Z* is the atomic number and *n* is between 4 and 5. On the other hand, the photoelectric cross-section is proportional to *1/E^3.5^*, where *E* is the energy of the radiation beam [15,16]. It is seen that the photoelectric enhancement is more significant, when the radiation beam contains more low-energy photons. Therefore, in using a different photon energy spectrum between the FF and FFF beam, the dose enhancement would be changed, when the FF is removed from the beam [17].

Recently, there are some studies regarding the depth dose enhancement, when heavy-atom NPs are added to an irradiated volume using the FF and FFF photon beams. These studies only considered a single beam energy or a specific normalization [18,19,20]. In the proposed phantom study, Monte Carlo simulation was carried out systematically to determine the dose enhancement varying with depth, when the FF was removed from the 6 and 10 MV photon beams. These two energies are the most popularly used in radiotherapy clinically [21].

The aim of this study is to investigate the increase of depth dose enhancement, when the FF is removed from the medical linear accelerator in gold NP-enhanced radiotherapy. Monte Carlo method is a mathematical algorithm based on random sampling to determine a numerical solution for a scientific problem. The accuracy of the Monte Carlo result depends on the number of history [22]. Therefore, an accurate and precise Monte Carlo simulation require a long computing time. Recently, with the advance of high-performance computing, highly accurate Monte Carlo simulation can be finished within a very short period (from days to hours) [23]. Monte Carlo simulation is well-known to be used in predicting the dose enhancement in gold NP-enhanced radiotherapy, designing and optimizing the heavy-atom NP radiosensitizer, and determining the DNA dosimetry and damage when NPs are added to a cancer cell [24,25,26].

## 2. Materials and Methods

### 2.1. Monte Carlo Simulation

**Figure 1:**
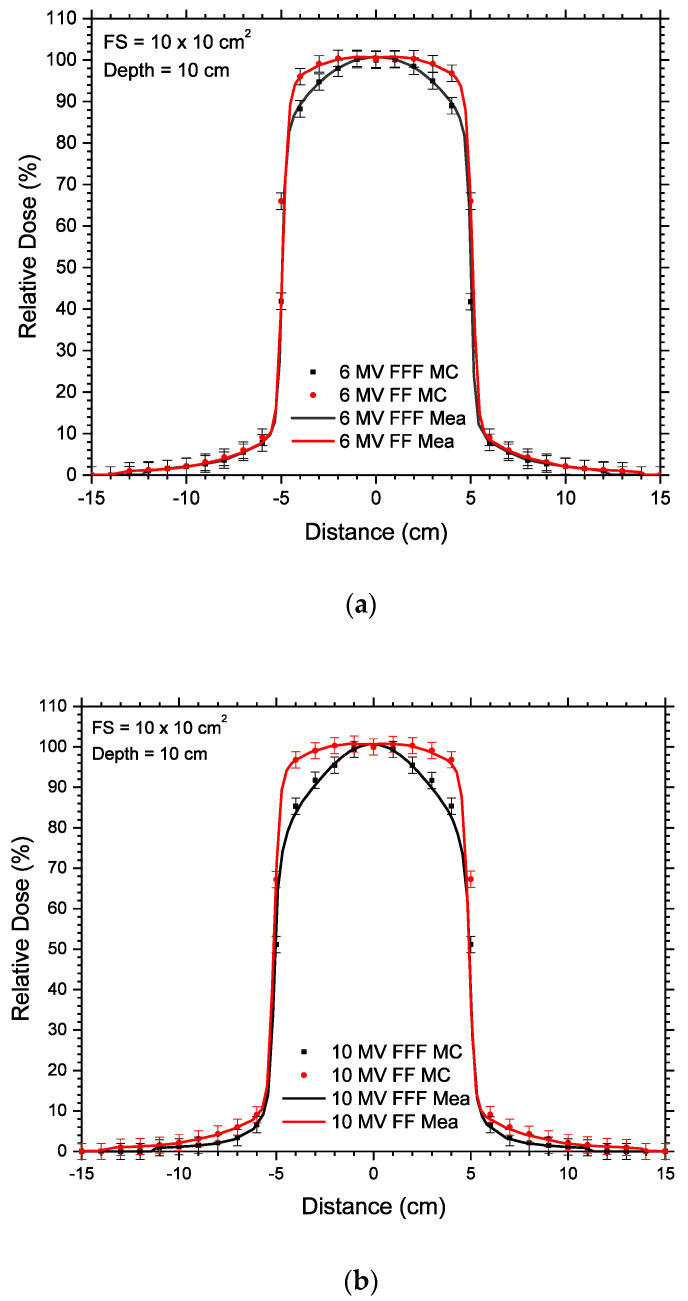
Off-axis profiles measured (Mea) and Monte Carlo simulated (MC) at a depth of 10 cm using the (**a**) 6 MV FF and 6 MV FFF; and (**b**) 10 MV FF and 10 MV FFF photon beams. The field size of the beam is equal to 10 × 10 cm^2^ used in this study.

The 6 and 10 MV FF and FFF photon beam models used in Monte Carlo simulation were based on a Varian TrueBEAM linear accelerator (Varian Medical System, Palo Alto, CA, USA). The photon beam energy spectra of the 6 MV FF, 6 MV FFF, 10 MV FF and 10 MV FFF beams were generated using the Geant4 (v4.9.2.p01) and EGSnrc-based BEAMnrc Monte Carlo codes [27,28]. The Geant4 simulation toolkit included a new discrete low-energy physics model for electron transport in gold with full atomic de-excitation cascade. Based on the information of configuration and materials of the accelerator head provided by the vendor, Monte Carlo model of the accelerator head from the beam source to the phase-space plane just above the jaws was created by the Geant4 code [29]. While the model of the accelerator head with the phase-space plane under the jaw was created by the EGSnrc-based BEAMnrc code [30]. This could take advantage of the component-module approach of BEAMnrc for modeling the jaw in the accelerator head. The field size of the beams was equal to 10 × 10 cm^2^, and there are 1 × 10^9^ particles on the phase-space plane. Verification of the Monte Carlo model was done by dosimetric measurements of percentage depth doses and beam profiles using scanning water tank and ionization chamber. Monte Carlo verifications for the percentage depth doses can be found elsewhere [30], while the beam profiles related to this study can be found in Figure 1a,b. The beam profiles in Figure 1a,b were normalized to 100% on the central axis. It is seen from Figure 1 that the agreement between the measured and Monte Carlo results was within 2%, showing that the simulation reproduced the measurement well.

**Figure 2:**
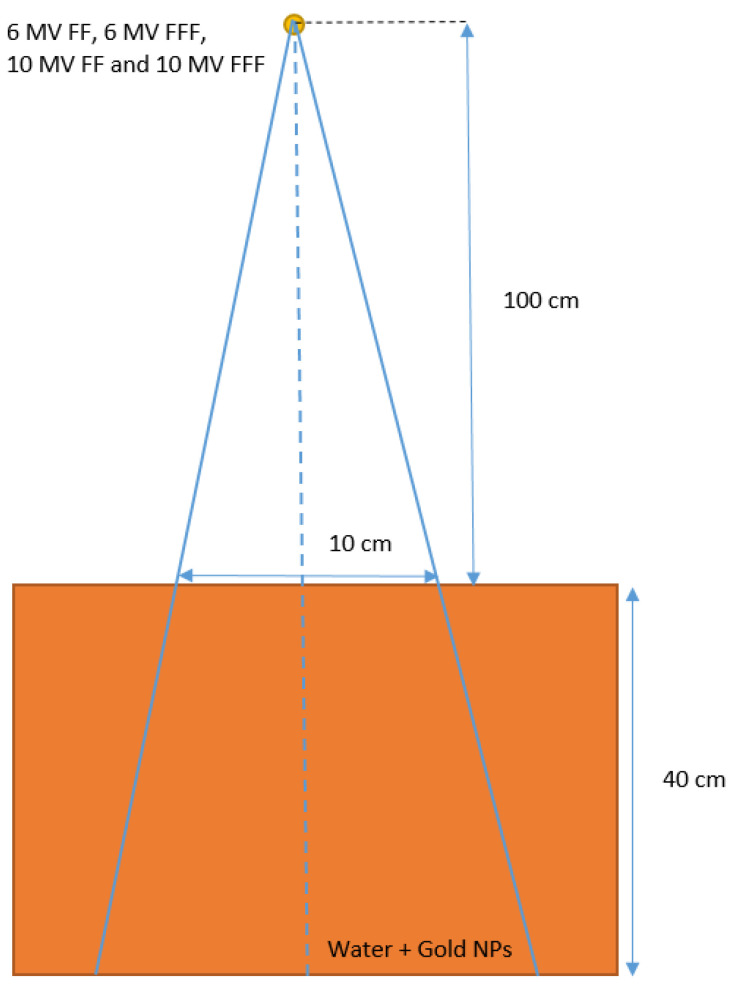
Schematic diagrams (not to scale) showing the heterogeneous phantom used in Monte Carlo simulations. The dimensions of the phantoms were equal to 40 × 40 × 40 cm^3^. The phantoms were irradiated by the 6 MV FF, 6 MV FFF, 10 MV FF and 10 MV FFF photon beams with field size equal to 10 × 10 cm^2^. The source-to-surface distance (SSD) was equal to 100 cm.

Figure 2 shows the simulation geometry in this study. The beam sources included the 6 MV FF, 6 MV FFF, 10 MV FF and 10 MV FFF photon beams. The source-to-surface distance (i.e., distance from the source to phantom surface) was equal to 100 cm. The material of the phantom was varied with pure water and a mixture of water and gold NPs at different concentrations in the range of 3–40 mg/mL as per studies [6,7,31,32]. In the simulation, the gold NPs were assumed to be distributed evenly in the phantom based on the material data library of NPs created using the EGSnrc-based PEGS code [6]. Depth doses up to 40 cm along the central beam axis (vertical broken line in Figure 2) in the phantom were determined using Monte Carlo simulation with variations of photon beams (6 and 10 MV FF and FFF) and NP concentrations (3–40 mg/mL). The simulations were carried out under the macroscopic approach using the EGSnrc Monte Carlo code [32,33], and therefore do not focus on the variables of NP shape and size. Sheeraz et al. [32] compared simulated DER due to addition of gold NPs irradiated by kilovoltage photon beams between the macroscopic and microscopic (i.e., considering the NP shape and size) approach. They found that the deviation between the two approaches was not significant. For megavoltage photon beams in this study, we will compare our results with those [34,35] using the microscopic approach in Section 4.1. The voxel size of the phantom in the simulation was equal to 1 × 1 × 1 cm^3^. The electron cut-off energy was set to 1 keV and the number of history in each simulation was set to 200 million.

### 2.2. Calculation of the Increase of Dose Enhancement Due to FF Removal

The increase of dose enhancement due to the removal of the FF from the photon beam can be determined by calculating the dose enhancement ratio (DER) and the FFF enhancement ratio (FFFER). The DER is defined as:

(1) $$\mathrm{DER}=\frac{\mathrm{Dose}\;\mathrm{at}\;a\;\mathrm{point}\;\mathrm{with}\;\mathrm{addition}\;\mathrm{of}\;\mathrm{gold}\;\mathrm{NPs}\;\mathrm{irradiated}\;\mathrm{by}\;a\;\mathrm{photon}\;\mathrm{beam}}{\mathrm{Dose}\;\mathrm{at}\;\mathrm{the}\;\mathrm{same}\;\mathrm{point}\;\mathrm{without}\;\mathrm{addition}\;\mathrm{of}\;\mathrm{gold}\;\mathrm{NPs}\;\mathrm{irradiated}\;\mathrm{by}\;\mathrm{the}\;\mathrm{same}\;\mathrm{photon}\;\mathrm{beam}}$$

In Equation (1), DER at a point inside the phantom is defined as the ratio of radiation dose with and without addition of gold NPs. Both dose points at the same position in the equation are irradiated by the same photon beam, that is, either the FF or FFF photon beam with the same energy. When DER is equal to 1, the addition of gold NPs to the phantom irradiated by a photon beam has no effect on the dose enhancement. To determine the increase of dose enhancement when the FF is removed from the accelerator head, the FFFER is calculated using the following expression:

(2) $$\mathrm{FFFER}=\frac{{\mathrm{DER}}_{\mathrm{FFF}}\;\mathrm{at}\;a\;\mathrm{dose}\;\mathrm{point}}{{\mathrm{DER}}_{\mathrm{FF}}\;\mathrm{at}\;\mathrm{the}\;\mathrm{same}\;\mathrm{dose}\;\mathrm{point}}$$

In Equation (2), DER_FFF_ is the DER at a dose point irradiated by a FFF photon beam, and DER_FF_ is the DER at the same point irradiated by the FF beam using the same energy. According to the photon beam energy spectrum, the FFF photon beam contains more low-energy photons than the FF beam [10]. This leads to a higher photoelectric enhancement and higher energy deposition at the dose point [15]. Considering only the same type of photon beams in the numerator and denominator (i.e., either FFF or FF), the FFFER value should be larger than one due to the addition of gold NPs in the phantom.

## 3. Results

**Figure 3:**
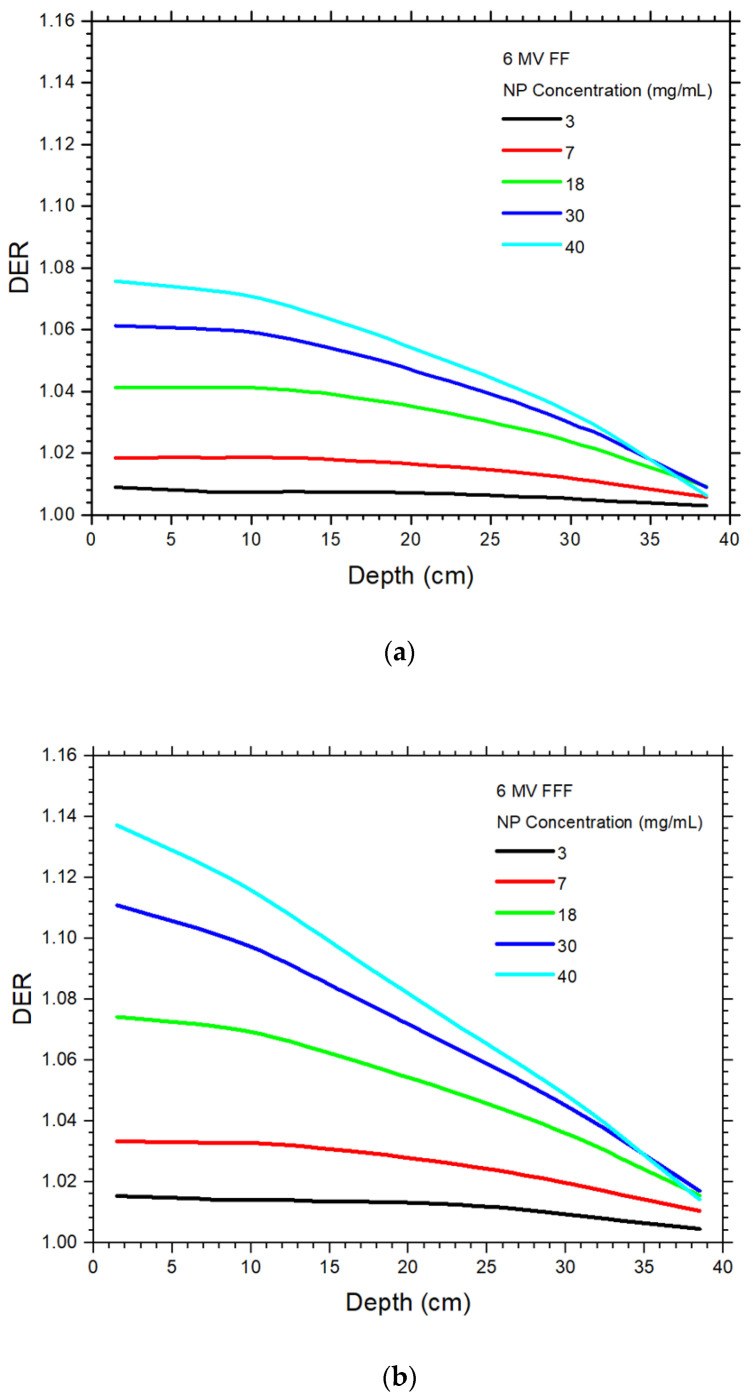
Relationships of dose enhancement ratio and phantom depth with variation of NP concentration using the (**a**) 6 MV FF and (**b**) 6 MV FFF photon beams.

**Figure 4:**
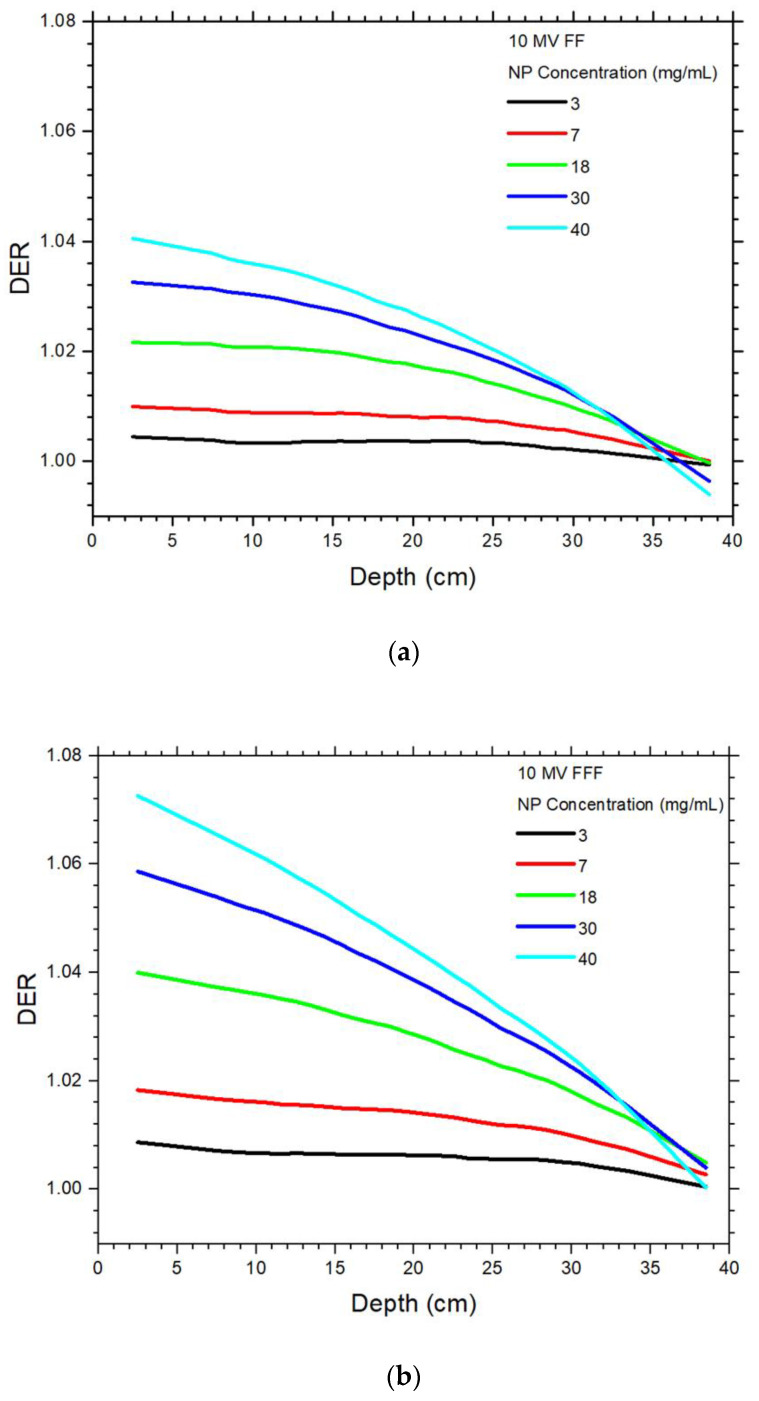
Relationships of dose enhancement ratio and phantom depth with variation of NP concentration using the (**a**) 10 MV FF and (**b**) 10 MV FFF photon beams.

**Figure 5:**
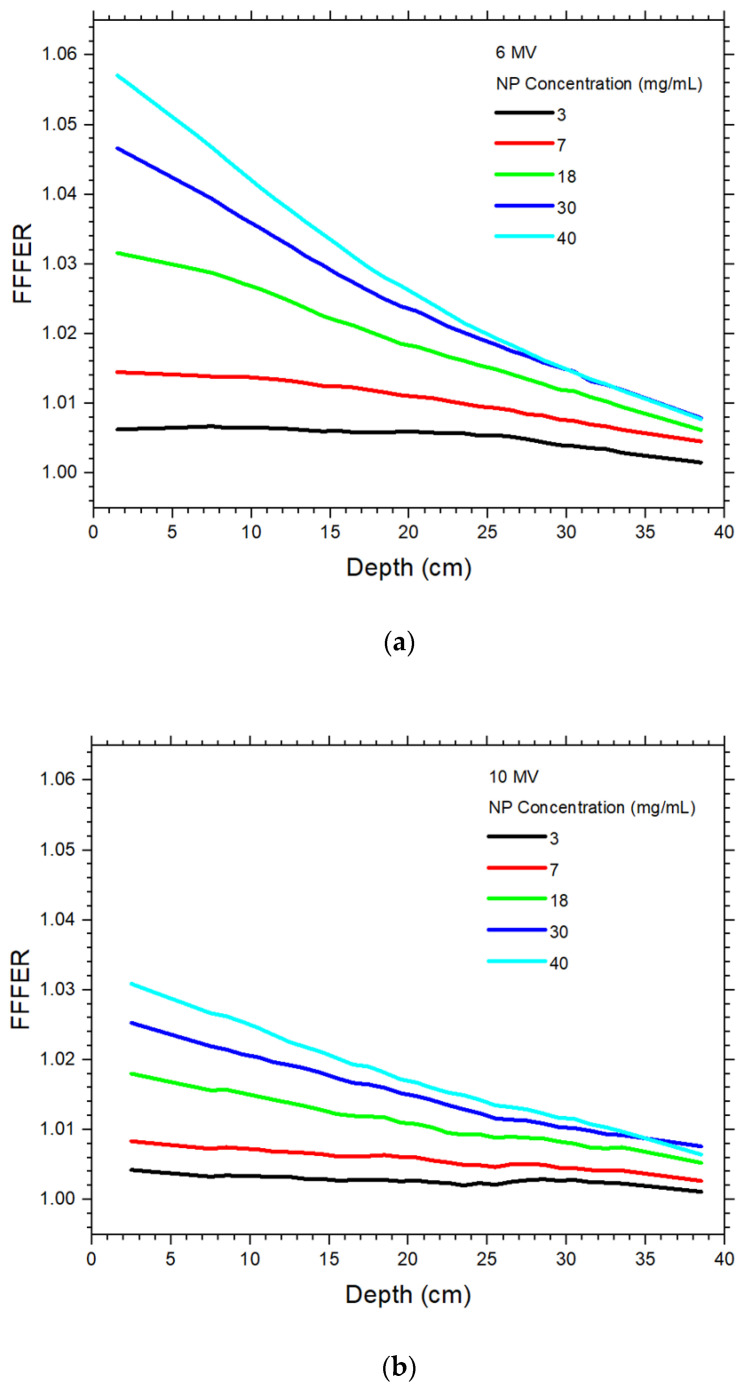
Relationships of flattening filter-free enhancement ratio and phantom depth with variation of NP concentration using the (**a**) 6 MV and (**b**) 10 MV photon beams.

The DER values of the 6 MV FF and FFF photon beams varying with the gold NP concentration are plotted against the phantom depth as shown in Figure 3a,b, respectively. The depth along the central beam axis was varied from 0 to 40 cm (Figure 2), while the NP concentration was set to 3, 7, 18, 30 and 40 mg/mL. Both figures are plotted using the same x and y scale for easy comparison. Using the same beam geometry and phantom material, Figure 4a,b show the DER values against the depth using the 10 MV FF and FFF photon beams, respectively. It should be noted that dose points in the build-up region of photon beams were unstable and not considered in this study, as deep-seated tumour in radiotherapy is usually found in the depth range of 5–30 cm [18]. It can be seen that DER values were larger than one in this depth range. The FFFER values of the 6 and 10 MV photon beam are plotted against the depth as shown in Figure 5a,b. The FFFER value reflected the increase of dose enhancement when the FF was removed from the accelerator head in gold NP-enhanced radiotherapy. Dependences of DER on the NP concentration and depth of phantom are shown in Table 1 for the 6 MV FF and 6 MV FFF photon beams. Similarly, Table 1 shows the dependences of DER on the NP concentration and depth of phantom for the 10 MV FF and 10 MV FFF photon beams. Dependences of FFFER on the NP concentration and depth of phantom are shown in Table 2 for the 6 and 10 MV photon beams.

## 4. Discussion

### 4.1. Dependences of Dose Enhancement on the Depth and NP Concentration

In Figure 3 and Figure 4, it is seen that the dose enhancement decreased from the surface to the bottom of the phantom for all NP concentrations and photon beams. The results agree with other studies regarding dose enhancements of depth dose using megavoltage photon beams [34,35,36]. For the 6 MV FF and 6 MV FFF beam, the DEF ranges in [34] were calculated as 1.00–1.09 and 1.01–1.19, while our corresponding results were 1–1.08 and 1.01–1.15. It should be noted that deviation between our results and [34] was expected as different beam variables, phantom geometry, NP concentration and simulation method (macroscopic vs. microscopic) were used in the study. For the 10 MV FF beam, the DER range was calculated using the microscopic approach as 1.01–1.03 in [35], which was close to our simulation results of 1.01–1.04. This decrease of DER values with depth as shown in Table 1 is due to the attenuation of low-energy photons along the central beam axis of the phantom. Considering the maximum DER values for the NP concentration of 40 mg/mL, the DER values were decreased 6.9, 12, 4.6 and 7.2% from phantom depth of 2 to 40 cm, for the 6 MV FF, 6 MV FFF, 10 MV FF and 10 MV FFF photon beams. The FFF photon beams decreased more significantly than the FF beams, because the FFF beams contained more low-energy photons than the FF beams [37]. This resulted in a higher attenuation of low-energy photons along the depth of phantom. For a specific depth in Figure 3 and Figure 4, the DER values increased with an increase of NP concentration. This is due to more gold NPs was added to the phantom, and therefore created a more significant photoelectric enhancement.

### 4.2. Dependence of Dose Enhancement on the Photon Beam Energy

For the 6 MV FF and FFF photon beams, it is seen that the DER values increased with an increase of NP concentration. The maximum DER values for the 6 MV FF and FFF photon beams were 1.08 and 1.14 for the NP concentration of 40 mg/mL (Figure 3a,b), while corresponding DER values for the 10 MV FF and FFF beams were 1.04 and 1.07, respectively. In Figure 3, the DER values increased with an increase of beam energy with respect to their beam type (i.e., FF and FFF beam). This is because the photon energy spectrum of the 6 MV beam had more low-energy photons than those of the 10 MV [10]. These low-energy photons contributed to the photoelectric enhancement, leading to more dose deposited at the depth of phantom and a higher DER value. Compared to the same beam type, the increases of DER value were 3.8% for the FF beam and 6.5% for the FFF beam, when the beam energy was decreased from 10 MV to 6 MV. It is found that the removal of FF also contributed to the dose enhancement change when the photon beam energy was decreased.

### 4.3. Dependence of Dose Enhancement on the Presence of FF

Considering the DER variation with depth using the same beam energy, it is seen in Figure 3 and Figure 4 that the DER value at the same dose point for the FFF beam was higher than the FF beam. For the maximum DER values in Figure 3 and Figure 4, the DER values were 5.7% (6 MV) and 3.1% (10 MV) higher between the FF and FFF photon beams. The higher DER values for the FFF beam is because when the FF was removed from the accelerator head, the low-energy photons, which should be attenuated by the filter, would remain in the FFF beam [37]. These low-energy photons were very sensitive to the photoelectric effect and hence produced a higher dose enhancement [16]. This phenomenon was true for both the 6 and 10 MV photon beams.

### 4.4. Dependence of the Increase of Dose Enhancement on the Absence of FF

Figure 5a,b show the FFFER values plotted against the depth for the 6 and 10 MV photon beams, respectively. It is seen that the FFFER values increased when the FF was removed from the photon beam. For both the 6 and 10 MV photon beams, the FFFER values decreased with an increase of depth in the phantom (Table 2). In addition, the higher the NP concentration, the higher the FFFER value. The maximum FFFER values at NP concentration of 40 mg/mL for the 6 MV photon beam was 1.057, which was larger than the 1.031 for the 10 MV beam. This reflects that the removal of FF from the photon beam increased the dose enhancement more significantly for the 6 MV photon beam compared to 10 MV, and the presence of FF played an important role in the dose enhancement apart from the beam energy and NP concentration. Moreover, the variation of FFFER with depth was more significant for the 6 MV beam than the 10 MV. This is due to the change of photon energy spectrum when the FF was removed from the accelerator head, resulting in more low-energy photons to be absorbed along the depth of phantom for the 6 MV beam [16].

In NP-enhanced radiotherapy, NPs can be localized in the tumour through intravenous or intratumoral injection. However, the NP concentration would vary from one tissue to another and even within the tumour itself. The simulation in this work assumes an even distribution of NPs and cannot address this aspect. Future work will be carried out regarding NPs distributed unevenly in a phantom.

## 5. Conclusions

Dose enhancement with variation of treatment depth due to addition of gold NPs was investigated using Monte Carlo simulation. The DER and FFFER varying with the photon beam energy, presence of FF and NP concentration were determined based on the Monte Carlo results of depth doses. It is found that the depth dose enhancement increased with the lower photon beam energy (6 MV), removal of FF (FFF beams) and an increase of NP concentration. When the FF was removed from the accelerator head, the FFFER results showed that the increase of dose enhancement of the 6 MV photon beam was higher than the 10 MV, when gold NPs were added to the phantom. Results in this study can help radiation staff to justify the dose distribution due to dose enhancement with addition of gold NPs, regarding the NP and beam variables, which are related to the depth dose enhancement in gold NP-enhanced radiotherapy.

## Figures and Tables

**Table 1:** Dependences of DER on the NP concentration and depth of phantom using the 6 MV FF, 6 MV FFF, 10 MV FF and 10 MV FFF photon beams.

	**6 MV FF**					**6 MV FFF**				
**Concentration** **(mg/mL)**	**3**	**7**	**18**	**30**	**40**	**3**	**7**	**18**	**30**	**40**
2 cm	1.0087	1.0185	1.0412	1.0611	1.0752	1.0150	1.0331	1.0736	1.1093	1.1347
5 cm	1.0080	1.0186	1.0413	1.0606	1.0737	1.0146	1.0329	1.0723	1.1049	1.1277
10 cm	1.0074	1.0187	1.0411	1.0588	1.0702	1.0140	1.0326	1.0687	1.0961	1.1142
15 cm	1.0074	1.0177	1.0387	1.0533	1.0624	1.0135	1.0304	1.0614	1.0833	1.0972
20 cm	1.0070	1.0163	1.0347	1.0461	1.0532	1.0130	1.0274	1.0534	1.0704	1.0801
25 cm	1.0062	1.0143	1.0294	1.0382	1.0434	1.0117	1.0238	1.0447	1.0574	1.0635
30 cm	1.0050	1.0115	1.0228	1.0285	1.0318	1.0089	1.0190	1.0348	1.0435	1.0466
35 cm	1.0037	1.0079	1.0144	1.0166	1.0162	1.0061	1.0135	1.0227	1.0272	1.0266
	**10 MV FF**					**10 MV FFF**				
**Concentration** **(mg/mL)**	**3**	**7**	**18**	**30**	**40**	**3**	**7**	**18**	**30**	**40**
2 cm	1.0044	1.0099	1.0215	1.0325	1.0405	1.0086	1.0183	1.0399	1.0586	1.0726
5 cm	1.0040	1.0095	1.0214	1.0318	1.0388	1.0077	1.0173	1.0383	1.0558	1.0683
10 cm	1.0032	1.0088	1.0207	1.0300	1.0356	1.0066	1.0160	1.0358	1.0510	1.0611
15 cm	1.0036	1.0087	1.0196	1.0271	1.0316	1.0063	1.0149	1.0321	1.0449	1.0525
20 cm	1.0035	1.0079	1.0171	1.0227	1.0262	1.0062	1.0139	1.0281	1.0379	1.0433
25 cm	1.0033	1.0071	1.0137	1.0179	1.0196	1.0054	1.0118	1.0227	1.0297	1.0333
30 cm	1.0050	1.0115	1.0228	1.0285	1.0318	1.0047	1.0095	1.0172	1.0216	1.0231
35 cm	1.0037	1.0079	1.0144	1.0166	1.0162	1.0022	1.0055	1.0099	1.0108	1.0092

**Table 2:** Dependences of FFFER on the NP concentration and depth of phantom using the 6 MV and 10 MV photon beams.

	6 MV					10 MV				
Concentration (mg/mL)	3	7	18	30	40	3	7	18	30	40
2 cm	1.0063	1.0143	1.0311	1.0454	1.0553	1.0042	1.0083	1.0179	1.0252	1.0308
5 cm	1.0065	1.0140	1.0297	1.0417	1.0502	1.0036	1.0076	1.0165	1.0232	1.0283
10 cm	1.0065	1.0136	1.0264	1.0352	1.0410	1.0033	1.0071	1.0147	1.0203	1.0245
15 cm	1.0060	1.0124	1.0218	1.0284	1.0327	1.0027	1.0061	1.0122	1.0173	1.0202
20 cm	1.0059	1.0109	1.0181	1.0232	1.0255	1.0027	1.0060	1.0108	1.0148	1.0167
25 cm	1.0054	1.0093	1.0148	1.0184	1.0193	1.0021	1.0046	1.0088	1.0116	1.0134
30 cm	1.0038	1.0074	1.0117	1.0145	1.0144	1.0028	1.0044	1.0079	1.0101	1.0115
35 cm	1.0023	1.0055	1.0081	1.0103	1.0102	1.0018	1.0035	1.0065	1.0085	1.0084

## Data Availability

Not applicable.
